# MetaMamba: Meta-learning with mamba for few-shot vegetation species classification using UAV-based hyperspectral imagery in desert rangeland

**DOI:** 10.1371/journal.pone.0352744

**Published:** 2026-08-31

**Authors:** Fei Hao, Xinchao Gao, Tao Zhang, Jianmin Du, Shengli Wang, Xiaoyan He

**Affiliations:** 1 Intelligent Manufacturing College, Hohhot Polytechnic University, Hohhot, Inner Mongolia, China; 2 College of Mechanical Engineering, Hebei Petroleum University of Technology, Chengde, Hebei, China; 3 College of Mechanical and Electrical Engineering, Inner Mongolia Agricultural University, Hohhot, Inner Mongolia, China; 4 School of Mechanical Engineering, Guiyang University, Guiyang, Guizhou, China; 5 Yangtze Delta Region Institute (Huzhou), University of Electronic Science and Technology of China, Huzhou, Zhejiang, China; Sultan Qaboos University, OMAN

## Abstract

Effective monitoring of desert rangeland ecosystems is of crucial significance to regional ecological security. Unmanned aerial vehicle (UAV) hyperspectral remote sensing provides an effective means for the fine identification of vegetation species. However, in practical applications, vegetation classification using hyperspectral images often faces the problem of limited labeled samples, which makes it difficult for traditional deep learning methods to obtain stable and accurate classification results. To address these issues, this study proposes a meta-learning with Mamba (MetaMamba) method for vegetation classification in desert rangeland. This method constructs a local-global dual-branch structure in the feature extraction stage to achieve effective fusion of local and global spatial context information. Specifically, the local branch uses convolutional neural networks (CNNs) to extract fine-grained spatial features, while the global branch models long-distance spatial dependencies based on the Mamba model. Additionally, a meta-learning strategy is introduced to enhance the feature learning and generalization abilities of the model under few-shot conditions. Experimental results show that the proposed method outperforms existing methods across multiple evaluation metrics. The overall classification accuracy (OA) reaches 90.85%, the average accuracy (AA) reaches 91.67%, and the Kappa coefficient reaches 87.77%. The method shows good stability and adaptability under different sample sizes. The MetaMamba model can achieve high-precision classification of desert rangeland vegetation species under few-shot conditions, providing an effective technical approach for ecological monitoring and rangeland resource management.

## Introduction

The desert rangeland is an important ecosystem type in Inner Mongolia, playing a crucial role in maintaining regional ecological security, ensuring animal husbandry production, and protecting biodiversity [1]. Vegetation is the core component of the desert rangeland ecosystem, and its species composition and spatial distribution patterns directly reflect the structure and function of the ecosystem [2]. In recent years, driven by multiple factors such as climate change and human activities, vegetation degradation in the Inner Mongolian rangeland has become increasingly prominent. This is manifested in reduced vegetation coverage, altered community structures, and the replacement of dominant species [3]. Therefore, it is of great significance to accurately obtain spatial distribution information on vegetation species to assess rangeland degradation and formulate scientific and reasonable ecological protection and restoration strategies.

Traditional vegetation species identification mainly relies on field surveys and manual interpretation to obtain information on vegetation composition and community structure [4]. Although these methods yield accurate species data, they are typically labor-intensive and time-consuming, and are constrained by terrain conditions and survey accessibility. Consequently, achieving efficient and continuous ecological monitoring across large scales remains challenging. With the advancement of remote sensing technology, vegetation identification using remote sensing data has gradually become a pivotal means of obtaining spatial distribution information [5,6]. Specifically, hyperspectral remote sensing captures continuous and rich spectral information, providing a robust basis for the fine differentiation between vegetation species [7,8]. In recent years, unmanned aerial vehicle (UAV) hyperspectral remote sensing has been widely adopted in rangeland vegetation monitoring and species identification due to its high spatial resolution, flexible acquisition, and ability to obtain detailed surface information at a local scale [9,10]. Compared with RGB or multispectral platforms, UAV hyperspectral data can more accurately characterize the spatial structure and spectral differences of vegetation patches, providing a more detailed dataset for species identification in the desert rangeland.

Early hyperspectral image classification methods mainly relied on traditional machine learning algorithms, which typically involved two stages: feature dimensionality reduction and classification. For example, common dimensionality reduction methods include principal component analysis (PCA) [11] and linear discriminant analysis (LDA) [12]; subsequently, support vector machine (SVM), random forest (RF), and K-nearest neighbor (KNN) models are frequently employed as classifiers [13,14]. To a certain extent, these methods utilize the spectral characteristics of hyperspectral data. However, due to inherent challenges—such as high dimensionality, band redundancy, and complex nonlinear relationships—traditional methods often rely on handcrafted features. Consequently, it is difficult to fully mine the potential spatial-spectral information within the data, thereby limiting classification performance. Furthermore, when dealing with high-dimensional features and limited training samples, the “curse of dimensionality” is prone to occurring, which further compromises classification accuracy.

Advances in deep learning technology have led to its widespread adoption in the field of hyperspectral image classification [15]. Compared with traditional machine learning methods, deep learning can automatically learn discriminative feature representations through multi-layered network architectures, thereby handling complex nonlinear relationships in hyperspectral data more effectively [16]. In recent years, researchers have proposed a variety of deep learning-based methods to identify vegetation types in the desert rangeland. For example, Zhu et al. [3] employed multi-scale convolution to identify degraded species, while Pi et al. [1] utilized 3D convolutional neural networks (3DCNN) to identify rangeland vegetation, improving recognition accuracy through parameter optimization. Furthermore, Wang et al. [17] developed a lightweight hybrid convolutional neural network tailored for edge devices to accurately identify vegetation species. Although these methods simultaneously leverage spectral information and spatial context, yielding superior classification results compared to traditional methods, they remain highly dependent on the availability of large-scale training samples [18]. Given that obtaining high-quality labeled samples in remote sensing is often costly and time-consuming, the application of deep learning methods in practical scenarios is frequently constrained [19]. Therefore, effectively exploiting discriminative features under the condition of limited labeled samples to further enhance classification performance remains a critical challenge in current research.

Given the limited availability of annotated samples, the field of few-shot learning has gradually adopted the meta-learning paradigm of “learning how to learn”. By training across multiple few-shot classification tasks, meta-learning enables models to acquire transferable prior knowledge and rapidly adapt to novel tasks with only a small number of labeled samples [20]. In recent years, meta-learning has also been progressively introduced into few-shot hyperspectral image classification. Liu et al. [21] employed a three-dimensional convolutional neural network to extract spectral–spatial features and integrated metric learning for few-shot classification. Zhang et al. [22] proposed a global prototype network that improves few-shot hyperspectral image classification performance by learning representative class prototypes. Gao et al. [23] utilized a deep relation network to model the nonlinear relationships between support samples and query samples. Li et al. [24] further extended meta-learning to cross-domain few-shot hyperspectral classification, enhancing the model’s transferability across different datasets. These studies demonstrate that meta-learning can effectively exploit limited labeled data through task-level training, providing a promising solution for alleviating the issue of insufficient samples in hyperspectral image classification. However, the application of meta-learning approaches to vegetation species classification in desert grasslands remains largely unexplored.

Meanwhile, hyperspectral images contain abundant spatial and spectral information, and how to effectively extract discriminative features from such complex data has become another critical factor affecting classification performance. Although convolutional neural networks (CNNs) exhibit strong local feature extraction capabilities, their ability to model long-range spatial dependencies and global contextual information is relatively limited due to the restricted receptive fields. In recent years, Mamba networks based on the state space model (SSM) have been increasingly applied to remote sensing image analysis due to their linear computational complexity and capability of modeling long-range dependencies. Chen et al. [25] proposed RSMamba, which extracts global spatial information from remote sensing images through multi-directional scanning strategies, demonstrating the potential of Mamba for remote sensing scene classification. For hyperspectral image analysis, Li et al. [26] introduced MambaHSI, which employs spatial Mamba and spectral Mamba to separately model spatial relationships and spectral band dependencies. He et al. [27] proposed IGroupSS-Mamba, which extracts multi-scale spatial–spectral features through interval grouping and multi-directional scanning mechanisms. Cao et al. [28] further applied Mamba to few-shot hyperspectral image classification and enhanced feature representation by integrating local and global features. These studies indicate that Mamba can effectively capture long-range dependencies in hyperspectral images, providing a novel feature extraction paradigm for obtaining more comprehensive spatial contextual information. Considering the complex spatial distribution patterns of vegetation in desert grasslands, this study introduces Mamba into the feature extraction network to complement the limitations of CNNs in global spatial relationship modeling.

Based on the aforementioned background, this study proposes a Mamba-based meta-learning (MetaMamba) method for vegetation classification of hyperspectral images. This method enhances the model’s generalization ability under few-shot conditions by integrating a dual-branch feature extraction network with meta-task learning. Specifically, a local-global dual-branch structure is designed for feature extraction: the local branch employs CNNs to extract fine-grained spatial features, while the global branch models long-distance spatial dependencies leveraging the Mamba model. By fusing the features from both branches, more comprehensive spatial context information is captured. Subsequently, a meta-learning strategy is implemented to perform similarity measurement and classification between samples, enabling the model to learn discriminative feature representations even with limited training data. The main contributions of this study are as follows:

(1) A novel Mamba-based meta-learning framework (MetaMamba) is proposed for hyperspectral vegetation classification, specifically designed to enhance feature learning and classification performance under few-shot conditions.
(2) A local-global dual-branch feature extraction architecture is developed. It integrates CNNs for capturing fine-grained local spatial features with the Mamba model for modeling long-range spatial dependencies, thereby effectively fusing multi-scale spatial context information.
(3) Comprehensive experiments were conducted on a UAV hyperspectral dataset of desert rangeland. The effectiveness and stability of the MetaMamba method are rigorously validated through comparison with state-of-the-art (SOTA) methods and extensive ablation studies.

## Materials and methods

### Study area

**Fig 1:**
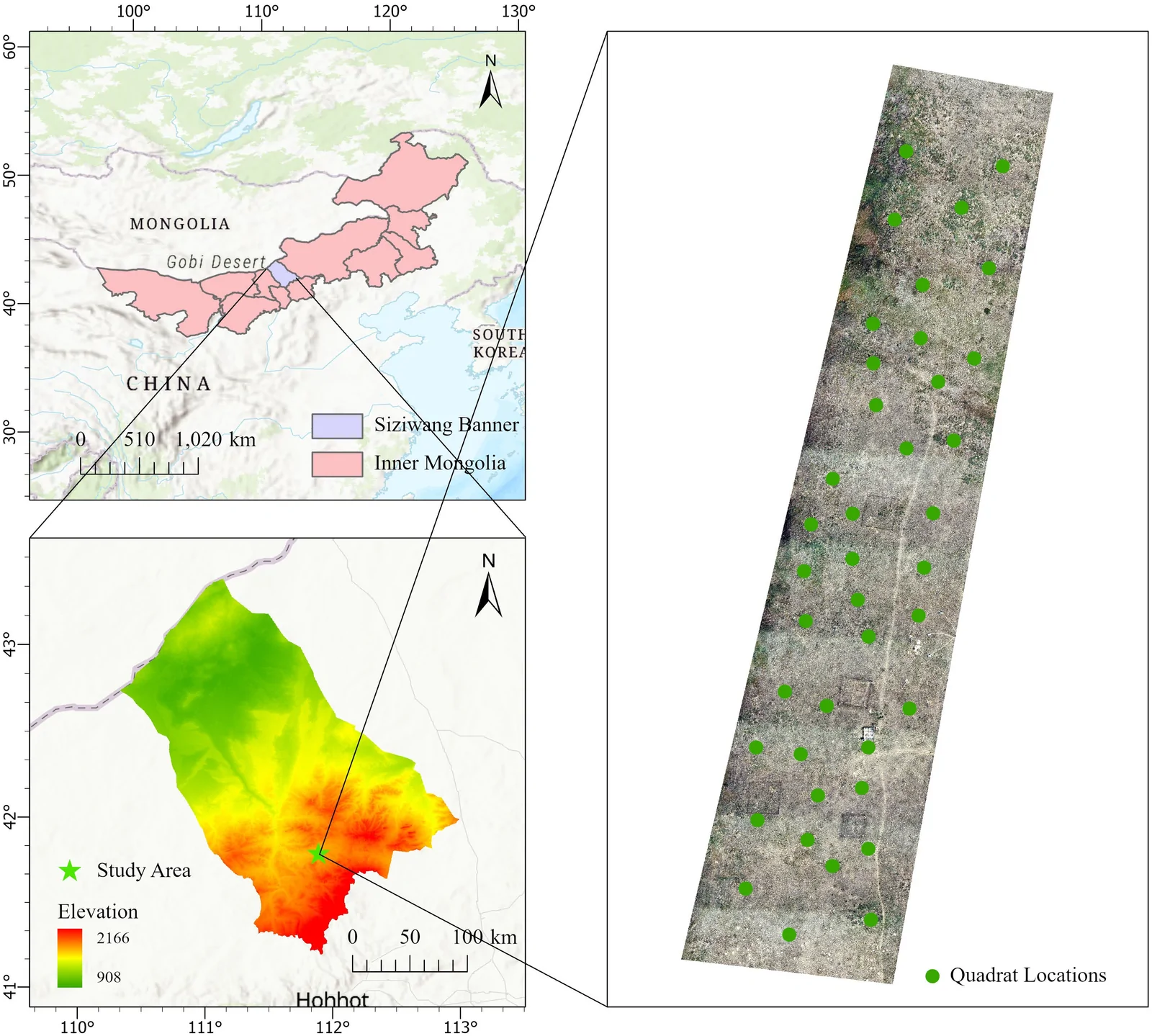
Geographical location of the study area.

The study area is located in the Gegentala rangeland of Inner Mongolia, China (Fig 1). The geographical coordinates are approximately 111°52′48″ E and 41°46′48″ N, with an average altitude of 1,450 m. Situated in the central part of the Inner Mongolia Plateau, the terrain is characterized by gentle slopes and low hills, representing a typical desert rangeland environment. The region features a mid-temperate continental climate, marked by cold, dry winters and warm summers with relatively concentrated precipitation. The annual average temperature ranges from 3°C to 4°C, and the annual precipitation is approximately 220–280 mm, most of which occurs during the growing season from June to September. Due to low precipitation and high evaporation, the regional climate exhibits distinct arid or semi-arid characteristics. The vegetation in the study area belongs to the typical *Stipa breviflora* desert rangeland community. The vegetation structure is relatively simple, characterized by low community height and sparse distribution. Vegetation coverage typically ranges from 15% to 25%, with an average height of 8–10 cm. The community is dominated by drought-tolerant herbs, among which *Stipa breviflora* is the primary constructive species, while *Cleistogenes songorica* and *Artemisia frigida* are common dominant species, and *Krascheninnikovia ceratoides* serves as a companion species.

### UAV hyperspectral image collection

In this study, a UAV hyperspectral remote sensing system was utilized to acquire vegetation hyperspectral imagery of the study area. The imaging system consists of a GaiaSky-mini hyperspectral camera, developed by Dualix Spectral Imaging. This sensor weighs 1.3 kg, featuring a lens with a 17 mm focal length. It captures data across 256 spectral bands within a wavelength range of 400–1000 nm, providing a spectral resolution of 3.5 nm. The camera is mounted on a DJI M600 Pro hexacopter platform and integrated with a high-precision gimbal stabilization system to ensure image stability during flight missions.

Field data collection was conducted in July during the peak growing season. Within the study area, 40 pure quadrats (1 × 1 m each) were established, with 10 quadrats per vegetation type. The study area covered approximately 4 hm^2^, and the spatial distribution of the 40 quadrats is shown in Fig 1. The central coordinates and specific vegetation types of each quadrat were recorded. The primary species sampled include typical desert rangeland plants such as *Stipa breviflora*, *Cleistogenes songorica*, *Artemisia frigida*, and *Krascheninnikovia ceratoides*. To ensure optimal image quality, data acquisition was performed between 10:00 and 14:00 (Beijing Time) under sunny, cloudless, and low-wind conditions to minimize the impact of varying illumination. During acquisition, the UAV flight altitude was set at 30 m, yielding hyperspectral images with a dimensions of 696 × 775 pixels and a spatial resolution of approximately 2.3 cm. Each region was captured at least three times to ensure data consistency. For radiometric calibration, standard black and white reference panels were utilized to perform reflectance correction before and after each flight, ensuring the acquisition of accurate surface reflectance. To convert the digital numbers (DNs) of the raw hyperspectral images into surface reflectance, images of black and white reference panels were acquired before and after each flight. Both the black and white reference panels were provided by Dualix Spectral Imaging Company as standard accessories of the hyperspectral imaging system. The white reference panel, with a nominal reflectance of 100%, was used to obtain the white reference signal. The black reference panel was used to acquire the dark reference signal and correct for sensor dark current and background noise. Additionally, a DJI Phantom 4 Pro was deployed to capture RGB imagery at an altitude below 5 m, providing high-definition visual references for dataset labeling and validation.

### Vegetation species dataset

Based on the ground survey results, the regions of interest (ROIs) were annotated using ENVI 5.3 software. By integrating the field-recorded sample locations and vegetation type information, corresponding ROIs were manually delineated on the hyperspectral imagery to label different land cover classes. The dataset comprises six classes: four primary vegetation species (*Stipa breviflora*, *Cleistogenes songorica*, *Artemisia frigida*, and *Krascheninnikovia ceratoides*) and two non-vegetation classes (Soil and Other). Specifically, the Other class mainly includes ground mats, small flags, and miscellaneous human-made litter. Following the completion of ROI annotation, pixel-level samples were extracted to construct the classification dataset. Ultimately, a total of 3,459 samples were obtained, as detailed in Table 1.

**Table 1:** Details of vegetation species dataset.

Class	Name	Samples
1	Stipa breviflora	1365
2	Other	244
3	Soil	284
4	Artemisia frigida	811
5	Cleistogenes songorica	401
6	Krascheninnikovia ceratoides	354

### Mamba-based meta-learning network

**Fig 2:**
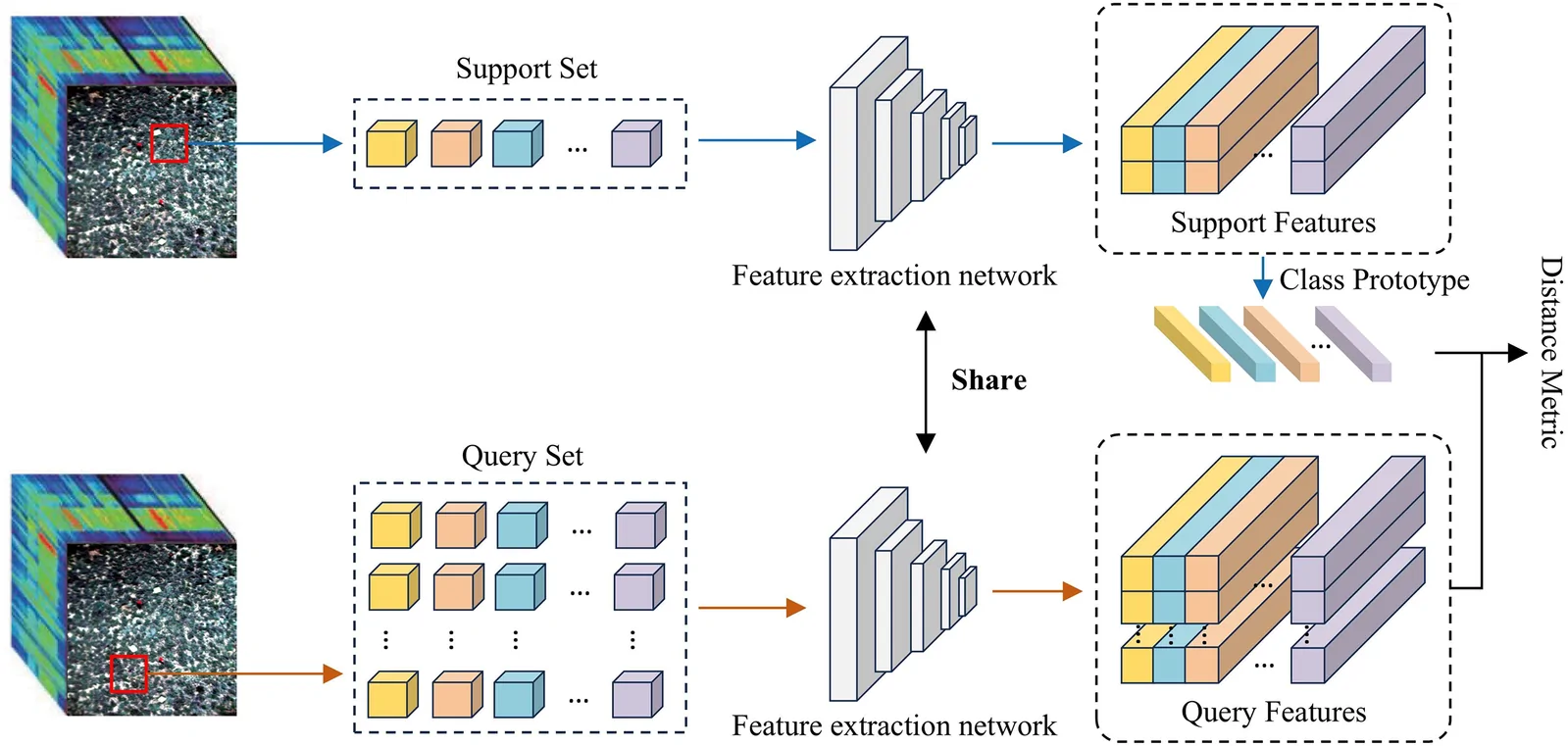
MetaMamba overall framework.

The overall framework of the proposed MetaMamba method is illustrated in Fig 2. Initially, spatial-spectral patches of size 7 × 7 × *L* are extracted from the hyperspectral imagery, centered on each target pixel, where *L* denotes the number of spectral bands. These patches are then partitioned into a support set and a query set to construct meta-tasks for model training. Subsequently, all patches are processed through a feature extraction network designed with a local-global dual-branch structure to learn highly discriminative feature representations. Finally, class prototypes are constructed based on the support set samples. The Euclidean distance between the query samples and these class prototypes is calculated as a similarity metric to perform final class prediction.

### Meta-learning

Under the meta-learning framework, the model is trained on a variety of meta-tasks to enhance its generalization capability under few-shot conditions. Each meta-task typically adopts a *C*-way *K*-shot learning configuration, where *C* denotes the number of classes in the task and *K* represents the number of labeled samples in the support set for each class. Within each meta-task, the data is partitioned into a support set and a query set. The support set is utilized to construct the feature representation for each class, while the query set serves to evaluate the model’s classification performance. The support set is defined as $S=\{(x_{i},y_{i}){\}}_{i=\text{1}}^{N_{s}}$, where $x_{i}$ represents the input sample, $y_{i}$ is the corresponding class label, and $N_{S}$ is the number of support samples. The query set is defined as $Q=\{(x_{j},y_{j}){\}}_{j=\text{1}}^{N_{q}}$, where $N_{q}$ denotes the number of query samples. Firstly, the support set and query set samples are input into the feature extraction network to obtain the corresponding feature representation. If the feature extraction function is F(·), then the feature representation of sample x can be expressed as Equation (1):


$$\begin{array}{c} z=F(x) \end{array}$$ (1)

After obtaining the feature embeddings of the support set, the class prototype for each class is constructed by calculating the mean vector of the features belonging to that class. For the *k*-th class, the class prototype $c_{k}$ is defined as Equation (2):


$$\begin{array}{c} c_{k}=\frac{\text{1}}{\left|S_{k}\right|}\sum_{x_{i}\in S_{k}}F\left(x_{i}\right) \end{array}$$ (2)

where $S_{k}$ denotes the set of support samples belonging to class (*k*), and $\left|S_{k}\right|$ represents the number of samples in that class, i.e., (*K*). Subsequently, for a query sample $x_{q}$, its feature representation is first obtained through the feature extraction network as $z_{q}=F\left(x_{q}\right)$. Then, the Euclidean distance between $z_{q}$ and the prototype of each class is calculated. The process is defined as Equation (3):


$$\begin{array}{c} d\left(z_{q},c_{k}\right)=∥z_{q}-c_{k}{∥}_{\text{2}} \end{array}$$ (3)

According to the distance, the probability that the query sample belongs to class *k* is calculated by Equation (4):


$$\begin{array}{c} p\left(y=k|x_{q}\right)=\frac{\text{exp}\left(-d\left(z_{q},c_{k}\right)\right)}{\sum_{k=\text{1}}^{C}\text{exp}\left(-d\left(z_{q},c_{k}\right)\right)} \end{array}$$ (4)

Finally, the model parameters are optimized by minimizing the cross-entropy loss function on the query set, as expressed in Equation (5):


$$\begin{array}{c} L=-\sum_{\left(x_{q},y_{q}\right)\in Q}\text{log}p\left(y=y_{q}|x_{q}\right) \end{array}$$ (5)

By training across a diverse set of meta-tasks, the model learns highly discriminative feature representations with robust generalization capabilities. This enables the effective identification of distinct vegetation classes even when provided with only a limited number of support samples, thereby overcoming the constraints of labeled data scarcity in desert rangeland monitoring.

### Mamba

Mamba is a sequence modeling approach based on the SSM, capable of effectively capturing long-distance dependencies while maintaining low computational complexity. Unlike traditional recurrent neural networks (RNNs) or Transformers, Mamba models sequential features through a continuous state space and achieves efficient computation with linear time complexity. Consequently, it demonstrates superior performance in processing long-sequence data, making it particularly suitable for capturing the complex spatial-spectral relationships inherent in hyperspectral imagery. In SSM, the dynamic changes of a sequence are usually described by Equations (6) and (7):


$$\begin{array}{c} h_{t}=𝐀h_{t-\text{1}}+𝐁x_{t} \end{array}$$ (6)

$$\begin{array}{c} y_{t}=𝐂h_{t} \end{array}$$ (7)

where $x_{t}$ represent the input feature at time *t*, $h_{t}$ denote the hidden state, and $y_{t}$ signify the output feature. The variables **A**, **B** and **C** are learnable parameter matrices that describe the state transition, input mapping, and output mapping, respectively. For a detailed derivation of the Mamba principles, please refer to the research by Gu et al. [29]. In the proposed method, the Mamba module is utilized to model the global relationships between different spatial positions within the hyperspectral image patches, thereby addressing the limitations of CNNs in capturing long-distance dependencies.

### Feature extraction network

**Fig 3:**
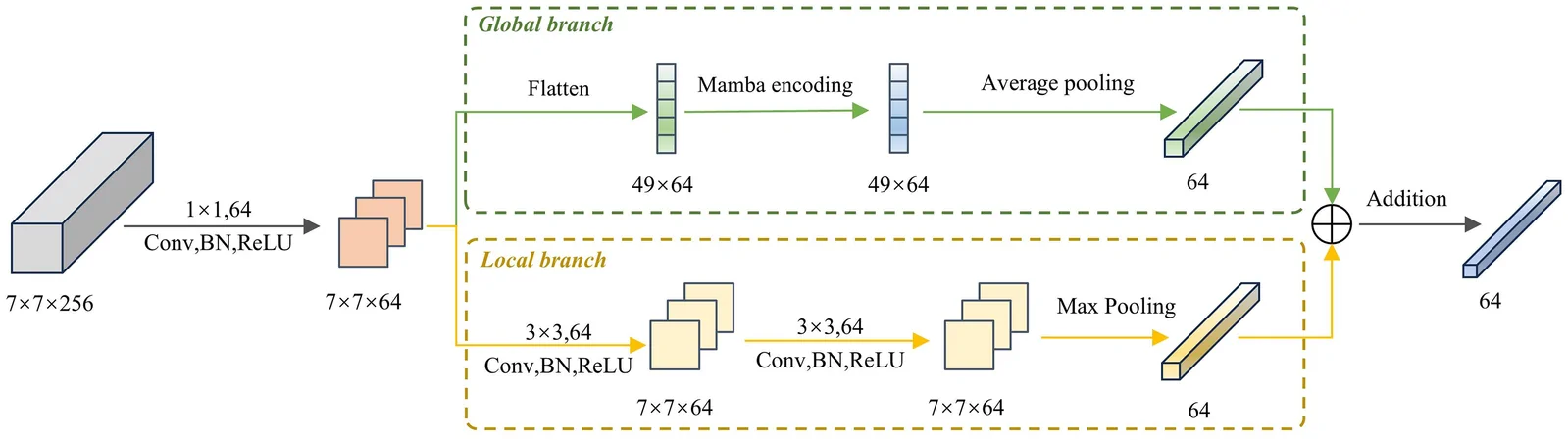
Feature extraction network.

To fully exploit the spatial context information within hyperspectral imagery, this study designs a local-global dual-branch feature extraction network. The architecture comprises three primary components: a shallow feature extraction module, a local branch (CNN), and a global branch (Mamba), as illustrated in Fig 3. By fusing local spatial features with global dependencies, the network generates more discriminative feature representations. Firstly, for the input hyperspectral image patch $x\in R^{\text{7}\times \text{7}\times L}$, shallow feature extraction is performed using 64 1 × 1 convolutional filters, as expressed in Equation (8):


$$\begin{array}{c} x_{\text{0}}=\sigma \left(\text{BN}\left(W_{s}*x\right)\right) \end{array}$$ (8)

where $W_{s}$ denotes the convolutional kernel parameters, BN(·) represents the batch normalization operation, σ(·) denotes the ReLU activation function. This operation linearly transforms the spectral dimensions while keeping the spatial structure intact, thereby obtaining a more compact feature representation and reducing the computational complexity for subsequent layers. Subsequently, the shallow features $x_{\text{0}}\in R^{\text{7}\times \text{7}\times \text{6}\text{4}}$ are fed into the local and global branches in parallel for concurrent feature extraction.

In the local branch, CNNs are used to extract fine-grained local spatial features. Specifically, neighborhood information is modeled using a two-layer 3 × 3 convolution operation. BN and ReLU functions are applied after each convolution layer to improve model stability. Finally, adaptive max pooling is used to aggregate the spatial features into a fixed-dimensional vector representation, as expressed in Equation (9):


$$\begin{array}{c} z_{l}=\text{MaxPool}\left(F_{l}\left(x_{\text{0}}\right)\right) \end{array}$$ (9)

where $F_{l}(\cdot )$ represents the convolution feature extraction function of the local branch, MaxPool(·) represents the max pooling function, and $z_{l}$ represents a local feature vector. This branch can effectively capture local spatial structure information between adjacent pixels in the patch.

In the global branch, the Mamba module is introduced to model a wider range of spatial dependencies. Firstly, the shallow features are expanded into a sequence form along the spatial dimension, resulting in $x_{seq}\in R^{\text{4}\text{9}\times \text{6}\text{4}}$. Then, the sequence is fed into the Mamba module for sequence modeling to capture long-range dependencies across different spatial locations. Finally, the global feature representation $z_{g}$ is obtained via global average pooling. The process can be expressed as Equation (10):


$$\begin{array}{c} z_{g}=\text{AvgPool}(\text{Mamba}\left(x_{seq}\right)) \end{array}$$ (10)

where Mamba(·) denotes the Mamba encoding function, and AvgPool(·) denotes the average pooling function. Compared to traditional convolution operations, Mamba can model long-range dependencies more effectively, thus alleviating the limitations of CNNs in global information modeling. After obtaining the local feature $z_{l}$ and the global feature $z_{g}$, the two features are fused to form the final feature representation, as expressed in Equation (11):


$$\begin{array}{c} z=z_{l}+z_{g} \end{array}$$ (11)

where z is the final output feature vector and is fed into the subsequent meta-learning classification module. With this local-global feature fusion mechanism, the model can simultaneously utilize fine-grained spatial structure information and long-range spatial dependencies, thereby improving its ability to discriminate vegetation.

### Experimental setup and evaluation metrics

The MetaMamba model is implemented using the PyTorch deep learning framework. The hardware configuration includes an Intel Core i7-11800H CPU, an NVIDIA GeForce RTX 3060 GPU (6 GB VRAM), and 16 GB of RAM. During model training, the learning rate is set to 0.001 over 100 episodes, utilizing the Adam optimizer for parameter updates. In the classification stage, similarity is measured by calculating the distance between the query samples and the class prototypes, with cross-entropy loss employed as the objective function for optimization.

Regarding data partitioning, five samples per class are randomly selected for training, while the remaining samples serve as the test set. To mitigate the challenge of limited training data, random radiation noise-based data augmentation was applied to the training samples to expand the training set to 200 samples per class. For the meta-learning configuration, meta-tasks are constructed following a *C*-way *K*-shot setting, where the support set is set to 1-shot and the query set consists of 19 samples.

To objectively evaluate classification performance, overall accuracy (OA), average accuracy (AA), and the Kappa coefficient are adopted as evaluation metrics. Specifically, OA represents the proportion of correctly classified samples across the entire test set; AA denotes the mean classification accuracy across all classes; and the Kappa coefficient measures the consistency between the classification results and random assignment. To minimize the impact of stochasticity, all experiments were repeated five times, with the average values reported as the final results.

## Results

### Patch parameter analysis

In order to analyze the impact of different patch sizes on model performance, we selected a variety of spatial scales for evaluation, and the results are shown in Table 2. The results show that patch size has a significant effect on the classification accuracy of hyperspectral vegetation species in desert rangeland. As the patch size increases from 3 × 3–7 × 7, the model performance continues to improve, with OA increasing from 80.90% to 90.85%, AA increasing from 83.60% to 91.67%, and Kappa increasing from 74.46% to 87.77%. This indicates that an appropriate increase in the spatial neighborhood can effectively utilize contextual spatial information around pixels and improve the model’s ability to discriminate different vegetation species. When the patch size further increases to 9 × 9 and 11 × 11, the classification accuracy does not improve further but fluctuates slightly. For example, the OA of 9 × 9 is 89.65%, lower than that of 7 × 7. Although the OA of 11 × 11 is 90.62%, the overall improvement is not obvious. At the same time, the standard deviation of large patch sizes increases, indicating reduced model stability. This suggests that an overly large spatial neighborhood may introduce more background information or heterogeneous pixels, thereby weakening the model’s ability to represent target class features to some extent. Overall, the 7 × 7 patch size delivers the best results across the three evaluation metrics while maintaining stable performance. This suggests that this scale strikes a good balance between exploiting spatial information and avoiding redundancy. Therefore, 7 × 7 is selected as the input size of the model in subsequent experiments.

**Table 2:** The effect of patch on model performance.

Patch	OA (%)	AA (%)	Kappa (%)
3 × 3	80.90 ± 1.69	83.60 ± 1.29	74.46 ± 2.24
5 × 5	87.30 ± 0.97	88.21 ± 0.58	83.09 ± 1.28
7 × 7	90.85 ± 1.75	91.67 ± 1.13	87.77 ± 2.36
9 × 9	89.65 ± 2.45	91.39 ± 1.10	86.39 ± 3.14
11 × 11	90.62 ± 2.96	91.87 ± 1.35	87.64 ± 3.84

### Ablation study

In order to analyze the influence of different branches in the feature extraction network on model performance, we conduct ablation experiments. It can be seen from Table 3 that the OA, AA, and Kappa of the model are 86.29%, 87.61%, and 81.94%, respectively, when only the local branch is used. This is because this branch mainly extracts local spatial features through convolution operations, which can effectively capture fine-grained information in the pixel neighborhood. However, its receptive field is limited, and its ability to model long-range dependencies and global structural information is relatively weak, resulting in certain limitations in overall classification performance. When only the global branch is used, the model performance is significantly improved, with OA, AA, and Kappa reaching 89.79%, 90.70%, and 86.55%, respectively. Compared with the case using only the local branch, the global branch shows significant improvements across all three metrics, indicating that Mamba-based feature extraction can more effectively model long-range dependencies and global contextual information, thereby improving the ability to distinguish different vegetation species. This demonstrates that global spatial structure information plays an important role in vegetation species identification in hyperspectral desert rangeland scenes. When both local and global branches are introduced simultaneously, the model achieves the best performance, with OA, AA, and Kappa reaching 90.85%, 91.67%, and 87.77%, respectively. Compared with single-branch configurations, the dual-branch structure further improves all three metrics, while the standard deviation is significantly reduced, indicating not only higher accuracy but also better stability. This shows that local and global features are highly complementary, where the local branch captures fine-grained spatial structures and the global branch models long-range dependencies and global contextual information. The combination of both branches enables more comprehensive characterization of spatial and spectral features in hyperspectral images, thereby improving overall vegetation classification performance in desert rangeland.

**Table 3:** Analysis of ablation experiment.

Local branch	Global branch	OA (%)	AA (%)	Kappa (%)
√		86.29 ± 4.00	87.61 ± 1.66	81.94 ± 5.03
	√	89.79 ± 3.40	90.70 ± 1.62	86.55 ± 4.38
√	√	90.85 ± 1.75	91.67 ± 1.13	87.77 ± 2.36

### Comparison with other methods

In order to verify the effectiveness of the MetaMamba method, we select seven state-of-the-art methods for comparative analysis, namely CGDC [30], CTAN [31], DIS-O [3], DNGNet [32], GDIF-3DCNN [1], SFMamba [33], and SGTN [16]. The parameter settings of these methods follow those reported in the original papers and are tested under the same experimental environment. In the comparative experiments, the same preprocessing and data augmentation strategies were adopted across all methods, ensuring a fair comparison. The results are presented in Table 4. The results show that the proposed MetaMamba method achieves the best performance, with OA, AA, and Kappa reaching 90.85%, 91.67%, and 87.77%, respectively, which are higher than those of all comparison methods. For example, compared with the better-performing SFMamba, the proposed method improves OA, AA, and Kappa by 2.35, 1.03, and 2.97 percentage points, respectively. Compared with CGDC, OA increases by 4.38 percentage points. This demonstrates that the proposed model has clear advantages in overall classification performance. In addition, the standard deviation is relatively small, indicating good model stability. From the per-class classification results, the proposed method achieves high recognition accuracy across most classes, with accuracy exceeding 80% for all classes. In particular, for class 1 (*Stipa breviflora*) and class 3 (Soil), the accuracy reaches 92.24% and 96.56%, respectively, significantly outperforming most comparison methods. This indicates that the proposed method can effectively extract highly discriminative features, thereby improving the recognition ability for complex vegetation types.

**Table 4:** Compares the results with other methods.

Class	CGDC	CTAN	DIS-O	DNGNet	GDIF-3DCNN	SFMamba	SGTN	MetaMamba (Ours)
1	80.53 ± 7.91	74.79 ± 5.10	49.96 ± 2.42	72.76 ± 7.31	61.06 ± 9.01	83.43 ± 2.90	55.50 ± 15.79	92.24 ± 2.04
2	86.19 ± 4.13	74.56 ± 1.17	80.67 ± 5.04	87.53 ± 4.80	83.68 ± 2.25	91.30 ± 3.15	90.54 ± 4.48	88.45 ± 2.87
3	61.29 ± 2.65	74.55 ± 7.64	83.51 ± 2.96	77.42 ± 7.29	60.86 ± 3.02	85.02 ± 14.49	97.06 ± 3.75	96.56 ± 4.18
4	97.97 ± 2.02	96.95 ± 1.10	71.32 ± 7.79	75.71 ± 12.36	85.66 ± 11.98	89.18 ± 6.23	76.08 ± 10.43	84.24 ± 5.72
5	92.32 ± 2.14	86.21 ± 1.45	78.23 ± 5.54	76.06 ± 25.10	83.99 ± 2.73	97.22 ± 1.25	82.37 ± 7.32	91.57 ± 1.89
6	96.73 ± 0.59	88.02 ± 3.74	93.52 ± 1.97	87.97 ± 6.44	70.77 ± 9.49	97.71 ± 2.15	75.36 ± 2.31	96.96 ± 1.71
OA (%)	86.47 ± 3.05	82.63 ± 2.49	67.55 ± 1.13	76.79 ± 4.10	72.04 ± 1.84	88.50 ± 0.61	71.29 ± 5.56	90.85 ± 1.75
AA (%)	85.84 ± 1.76	82.51 ± 2.31	76.20 ± 0.73	79.57 ± 5.54	74.34 ± 1.58	90.64 ± 1.82	79.49 ± 2.69	91.67 ± 1.13
Kappa (%)	82.10 ± 3.90	77.16 ± 3.19	59.09 ± 1.30	69.18 ± 6.07	63.53 ± 1.94	84.80 ± 0.89	63.14 ± 6.46	87.77 ± 2.36

**Fig 4:**
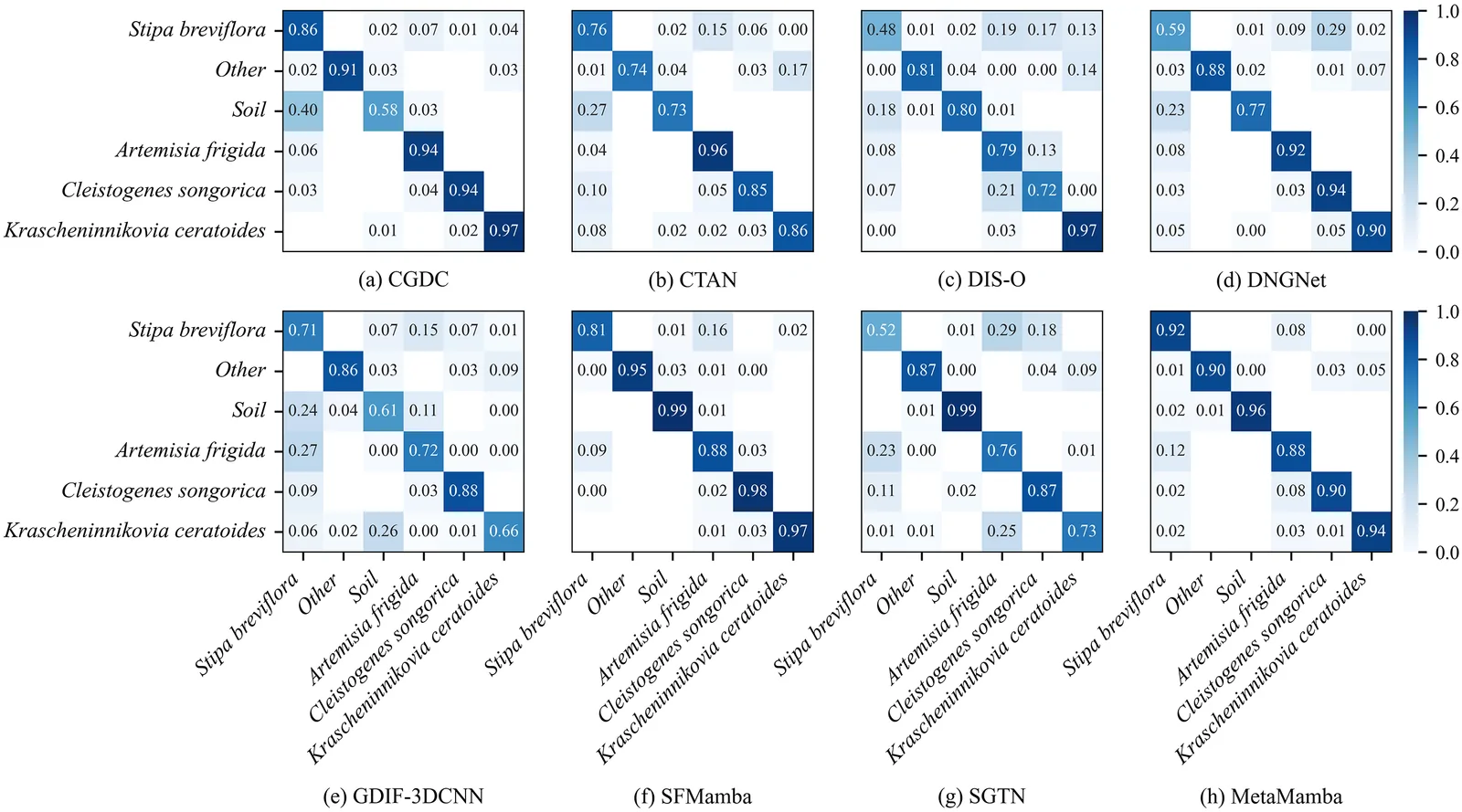
Confusion matrix results of different methods.

From the confusion matrix shown in Fig 4, it can be observed that there are significant differences in the classification performance of different methods across various classes, and the proposed MetaMamba method demonstrates more robust and accurate performance. In general, most comparison methods exhibit obvious confusion between certain vegetation classes, such as *Stipa breviflora* and Soil, and some classes are also easily misclassified as *Artemisia frigida*. For example, in methods such as CGDC, DIS-O, and GDIF-3DCNN, misclassification of *Stipa breviflora* into Soil or *Artemisia frigida* is more frequent, indicating that these methods still face challenges in distinguishing these classes. In contrast, MetaMamba shows higher diagonal elements in most classes, indicating improved classification accuracy. Specifically, *Stipa breviflora*, Other, *Cleistogenes songorica*, and *Krascheninnikovia ceratoides* achieve high recognition rates. Meanwhile, the number of off-diagonal elements in each class is significantly reduced, indicating that inter-class confusion is effectively alleviated. Although a small number of misclassifications still exist in the *Artemisia frigida* class, the overall error rate remains low. These results demonstrate that the proposed method can more effectively extract discriminative spatial-spectral features, thereby improving the ability to distinguish vegetation species in desert rangeland.

### Feature visualization

**Fig 5:**
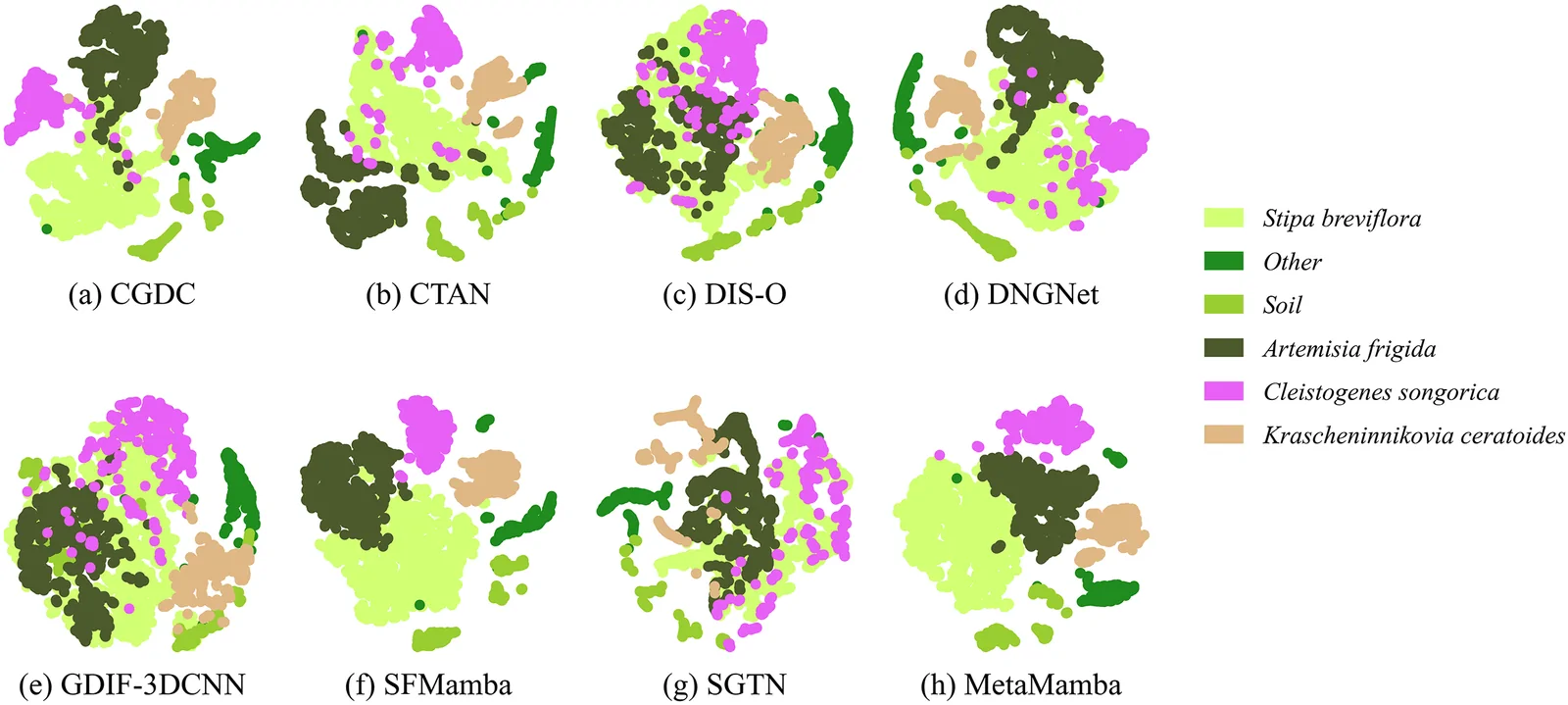
Feature visualization results of different methods.

The t-SNE visualization method is used to visualize the features extracted by each model. It can be seen from Fig 5 that the feature distributions of several comparison methods are highly dispersed, with significant overlap between different classes. For example, in the feature spaces of CGDC, CTAN, and DIS-O, the boundaries between different vegetation classes are blurred, and some samples are mixed together, indicating that these methods still have limitations in learning discriminative features. Although DNGNet and GDIF-3DCNN form relatively compact clusters for some classes, a certain degree of overlap between different classes can still be observed, indicating that their feature discriminability still has room for improvement. In contrast, the feature distributions of SFMamba and SGTN are more compact, and the separation between some classes is improved; however, the boundaries of certain classes are still not sufficiently clear. By contrast, the proposed MetaMamba method shows a clearer class structure in the feature space. Samples from each class form compact and well-separated clusters in the two-dimensional embedding space, and the overlap between different classes is significantly reduced. In particular, the feature distributions of several main vegetation types are more concentrated, with more distinct inter-class separation. This demonstrates that MetaMamba can learn more discriminative feature representations, thereby effectively improving the ability to distinguish between different vegetation species, which is consistent with the aforementioned quantitative results.

### Computational complexity analysis

In order to comprehensively evaluate the computational complexity of MetaMamba and comparison methods, we consider model parameters, inference time, and floating-point operations (FLOPs) for comparative analysis. The results are shown in Table 5. In terms of inference time, most methods exhibit short running times on the test set. CTAN and DIS-O have inference times of 0.28s and 0.29s, respectively, which are the fastest among all comparison methods. The proposed MetaMamba method has an inference time of 0.37s. Although slightly higher than some lightweight models, it is still significantly faster than DNGNet (2.03s), GDIF-3DCNN (1.07s), and SGTN (1.44s). This indicates that the proposed model achieves good inference efficiency while maintaining high classification accuracy. In terms of computational complexity, the FLOPs of MetaMamba are 12.12 M, which is lower than those of CGDC, GDIF-3DCNN, SFMamba, and SGTN, and only slightly higher than that of DIS-O and other lightweight models. This indicates that the overall computational cost of the model is well controlled. In terms of parameters, MetaMamba has 0.12 M parameters, which is comparable to SGTN and GDIF-3DCNN, and much lower than CGDC (1.13 M) and SFMamba (0.9 M). Overall, MetaMamba achieves better classification performance while maintaining lower model complexity and faster inference speed, demonstrating a good balance between efficiency and accuracy.

**Table 5:** The results of computational complexity analysis of different methods.

Metrics	CGDC	CTAN	DIS-O	DNGNet	GDIF-3DCNN	SFMamba	SGTN	MetaMamba (Ours)
Time (s)	0.53	0.28	0.29	2.03	1.07	0.33	1.44	0.37
FLOPs (M)	45.77	17.2	2.7	15.37	43.86	16.35	67.77	12.12
Params (M)	1.13	0.11	0.32	0.08	0.11	0.9	0.12	0.12

### Analysis of different numbers of training samples

**Fig 6:**
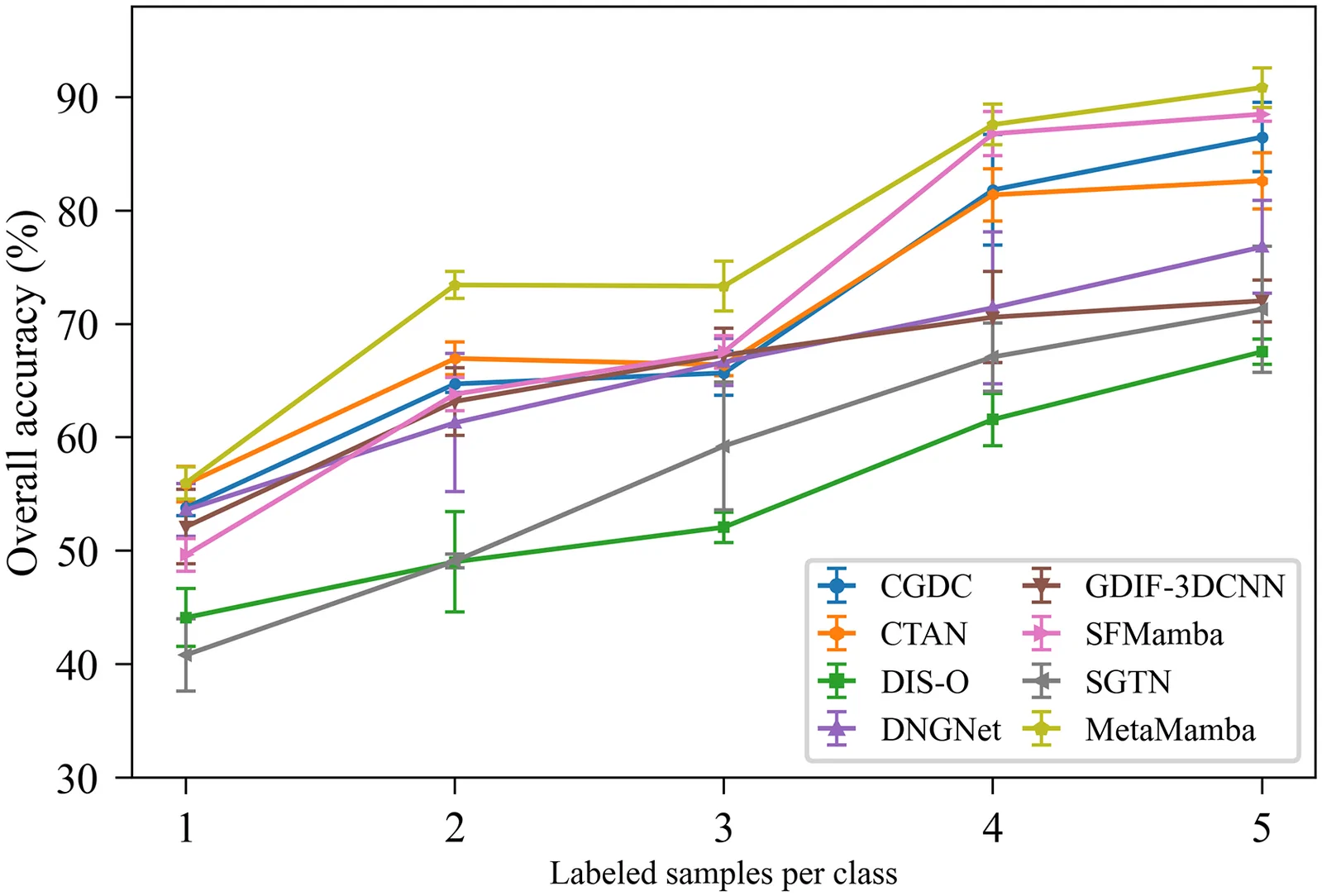
Performance of various methods under different numbers of training samples.

In order to further analyze the performance advantages of MetaMamba under few-shot conditions, 1, 2, 3, 4, and 5 samples per class are selected as training samples to compare the accuracy variations of each method under limited data conditions. According to the results shown in Fig 6, as the number of training samples increases, the classification accuracy of all methods shows an upward trend, indicating that more labeled samples can effectively improve the learning ability of the models. However, there are significant differences in the sensitivity of different methods to changes in sample size. For example, DIS-O and SGTN perform poorly when the number of samples is small, and achieve low accuracy with only 1 or 2 samples per class, which gradually improves as the number of samples increases. CGDC, CTAN, and DNGNet exhibit relatively stable performance under few-shot conditions, but their overall accuracy improvement is limited. In contrast, the proposed MetaMamba consistently achieves the best performance under all sample sizes and maintains the highest classification accuracy. Moreover, as the number of samples increases, the improvement in MetaMamba’s accuracy becomes more pronounced. At the same time, MetaMamba shows smaller error bars, indicating lower variance and better stability across different experimental runs. These results further demonstrate that the proposed method has stronger feature learning and generalization ability in few-shot scenarios, and can effectively improve the classification performance of vegetation species in desert rangeland under limited labeled data conditions.

### Generalization analysis

To further validate the generalization capability of the proposed model, the Salinas dataset was selected as the target dataset for generalization analysis. The Salinas dataset is a widely used agricultural scene benchmark dataset in hyperspectral image classification, collected by the AVIRIS sensor over the Salinas Valley, California, USA. The image size is 512 × 217 pixels, and the original data contain 224 spectral bands with a spatial resolution of approximately 3.7 m. After removing water absorption bands, 204 effective bands are typically retained, with annotated samples covering 16 land-cover classes. All experiments were conducted using the same preprocessing strategy and training repetitions to ensure a fair comparison.

According to the results in Table 4, the three comparison methods with the top overall performance (CGDC, CTAN, and SFMamba) were selected for comparison with the proposed MetaMamba. As shown in Table 6, MetaMamba achieved the best performance across the overall evaluation metrics, with OA, AA, and Kappa reaching 86.85%, 90.86%, and 85.32%, respectively. Compared with the closest-performing method, SFMamba, MetaMamba improved these three metrics by 0.46%, 0.42%, and 0.50%, respectively. Meanwhile, MetaMamba also demonstrated clear advantages over CGDC and CTAN. More importantly, MetaMamba achieved the lowest standard deviations among the three overall metrics, with values of 0.46, 0.22, and 0.50, respectively, indicating more stable generalization performance across repeated experiments.

**Table 6:** Performance comparison on the Salinas dataset.

Class	CGDC	CTAN	SFMamba	MetaMamba (Ours)
Brocoli_green_weeds_1	80.93 ± 28.28	61.68 ± 30.89	95.98 ± 3.92	97.72 ± 1.56
Brocoli_green_weeds_2	99.83 ± 0.18	99.19 ± 0.24	98.00 ± 3.91	99.55 ± 0.41
Fallow	68.84 ± 15.17	46.40 ± 17.06	66.21 ± 10.94	67.91 ± 2.93
Fallow_rough_plow	99.01 ± 0.19	96.95 ± 1.26	97.98 ± 1.69	99.22 ± 0.56
Fallow_smooth	95.70 ± 1.36	91.25 ± 1.79	98.88 ± 1.00	92.88 ± 1.84
Stubble	99.94 ± 0.05	99.76 ± 0.20	100.00 ± 0.00	100.00 ± 0.00
Celery	98.85 ± 0.36	98.90 ± 0.41	99.56 ± 0.27	98.96 ± 0.68
Grapes_untrained	81.89 ± 7.54	79.82 ± 3.82	81.41 ± 3.44	84.12 ± 3.07
Soil_vineyard_develop	98.01 ± 2.17	95.33 ± 1.21	98.08 ± 1.90	99.17 ± 0.40
Corn_senesced_green_weeds	76.74 ± 2.80	66.77 ± 6.39	76.63 ± 8.23	72.92 ± 0.86
Lettuce_romaine_4wk	94.79 ± 1.90	87.58 ± 2.77	91.23 ± 5.27	95.67 ± 1.91
Lettuce_romaine_5wk	99.83 ± 0.12	99.09 ± 1.31	95.63 ± 3.71	99.98 ± 0.04
Lettuce_romaine_6wk	99.69 ± 0.25	99.32 ± 0.21	99.25 ± 0.84	99.30 ± 0.38
Lettuce_romaine_7wk	95.74 ± 1.01	97.30 ± 0.42	96.79 ± 1.10	93.95 ± 1.44
Vineyard_untrained	45.84 ± 14.87	44.98 ± 14.96	56.21 ± 10.15	54.73 ± 3.23
Vineyard_vertical_trellis	97.46 ± 1.88	91.17 ± 2.64	95.17 ± 4.24	97.75 ± 0.40
OA (%)	84.85 ± 1.01	81.19 ± 1.44	86.39 ± 0.68	86.85 ± 0.46
AA (%)	89.57 ± 1.23	84.72 ± 2.02	90.44 ± 0.99	90.86 ± 0.22
Kappa (%)	83.08 ± 1.13	79.01 ± 1.64	84.82 ± 0.77	85.32 ± 0.50

From the perspective of class-wise accuracy, MetaMamba achieved the highest or near-highest classification accuracy on several categories, including Brocoli_green_weeds_1, Fallow_rough_plow, Grapes_untrained, Soil_vineyard_develop, Lettuce_romaine_4wk, Lettuce_romaine_5wk, and Vineyard_vertical_trellis. In particular, for categories with relatively large performance fluctuations, such as Brocoli_green_weeds_1, Fallow, and Vineyard_untrained, MetaMamba significantly reduced the standard deviation, demonstrating its ability to more stably adapt to inter-class spectral variations and cross-domain distribution shifts. Although MetaMamba achieved slightly lower accuracy than the best-performing comparison methods on a few categories, including Fallow_smooth, Corn_senesced_green_weeds, and Lettuce_romaine_7wk, it still exhibited superior overall accuracy and stability. These results demonstrate that the proposed method not only maintains competitive classification performance but also possesses stronger cross-scene generalization capability.

### Classification results of the sample plots

**Fig 7:**
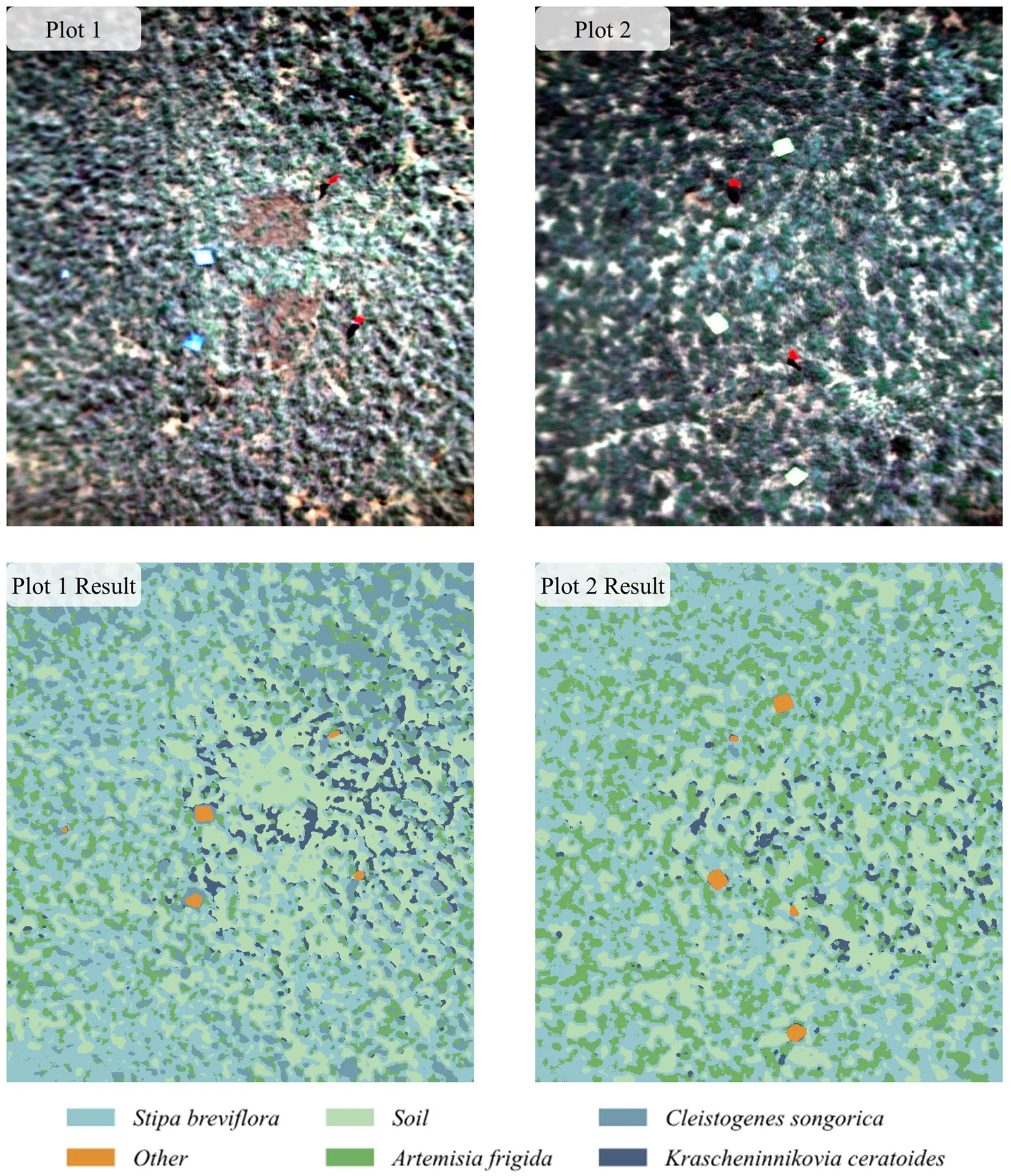
Visual analysis of classification results.

In order to further evaluate the classification performance of MetaMamba in practical applications, we selected two representative plots within the study region for visualizing the classification results, as shown in Fig 7. The results show that MetaMamba can better capture the spatial distribution characteristics of vegetation in desert rangeland, with different classes exhibiting clear spatial patch structures. In plot 1, *Stipa breviflora* and *Artemisia frigida* are the dominant vegetation types, accompanied by a certain proportion of Soil and a small number of Other classes. The classification results are consistent with the vegetation patch distribution in the original image, and the overall spatial structure appears more continuous. In plot 2, the model can effectively identify vegetation types such as *Cleistogenes songorica* and *Krascheninnikovia ceratoides* while maintaining good spatial integrity. The distribution of classified patches is largely consistent with the vegetation patterns in the image. From a visual perspective, most regions in the classification map exhibit coherent spatial structures, with only a small number of scattered pixels remaining at class boundaries, which is a common phenomenon in highly heterogeneous desert rangeland environments. Overall, MetaMamba can accurately describe the spatial distribution patterns of different vegetation types, demonstrating its good applicability in real-world scenarios.

## Discussions

The vegetation species identification in desert rangeland is extremely challenging. The proposed MetaMamba in this study achieves better performance than existing methods on the vegetation species classification task in desert rangeland, mainly due to the effective combination of model architecture design and task characteristics.

In the feature extraction stage, the network adopts a dual-branch structure consisting of local and global branches, enabling the model to simultaneously capture fine-grained local information and long-range spatial dependencies. Traditional CNN-based methods mainly rely on local convolution operations for feature extraction. Although they can effectively model local spatial structures, their receptive field is limited, making it difficult to fully capture large-scale spatial context information. The global branch based on Mamba can model long-range dependencies with low computational complexity, thereby better describing the spatial structure and distribution patterns of vegetation patches. After feature fusion, the two types of features form a more discriminative spatial-spectral representation, which is also an important reason why the dual-branch structure outperforms the single-branch setting in the ablation study.

In addition, local and global features are highly complementary. In desert rangeland ecosystems, vegetation species are usually distributed in patchy patterns, and different species may exhibit similar spectral characteristics. For example, some shrubs and herbs may show similar spectral signatures in hyperspectral images, making them difficult to distinguish using only single-pixel or local features. The local branch can extract fine-grained textures and local spatial structures within vegetation patches, while the global branch can utilize larger-scale spatial context information to describe vegetation community structure, thereby effectively alleviating spectral confusion among different species. This is also supported by the confusion matrix and t-SNE visualization results. The features learned by MetaMamba exhibit more compact and clearly separated class distributions in the feature space.

It is worth noting that MetaMamba maintains high classification accuracy under few-shot conditions, which is closely related to the introduction of the meta-learning framework. Vegetation surveys in desert rangeland typically require extensive field sampling, making it difficult to obtain large-scale labeled datasets. Therefore, the model must possess strong few-shot learning capability. Through the meta-learning training strategy, the model can learn shared feature representations across multiple tasks, thereby improving its adaptability to unseen classes. As a result, MetaMamba maintains relatively stable performance under extremely limited samples and shows more pronounced improvements as the number of samples increases.

In addition, in the parameter analysis, it is observed that patch size has a significant impact on model performance. When the patch size increases from 3 × 3–7 × 7, classification accuracy improves significantly, indicating that an appropriately larger spatial neighborhood helps the model utilize richer contextual information and alleviate spectral mixing and noise issues commonly present in hyperspectral data. However, when the patch size further increases, performance no longer improves and may even fluctuate. This may be because overly large neighborhoods introduce more background information or heterogeneous pixels, increasing class confusion, especially in desert rangeland environments where vegetation distribution is highly heterogeneous. Therefore, a 7 × 7 patch size achieves a good balance between spatial information utilization and redundant information.

Although the proposed MetaMamba achieves strong performance in hyperspectral vegetation classification in desert rangeland, there are still some limitations. On the one hand, this study mainly uses spatial and spectral information from hyperspectral images and does not incorporate additional auxiliary data (such as LiDAR, high-resolution RGB, or multi-temporal remote sensing data), which to some extent limits further exploration of complex vegetation structural information. Future work will focus on integrating multi-source remote sensing data to further improve the model’s ability to characterize vegetation structure and growth dynamics. In addition, although the meta-learning framework improves performance under few-shot conditions, model stability may still degrade under extremely low sample scenarios or more complex vegetation distributions. Therefore, more advanced modeling strategies could be explored in the future to enhance adaptability in complex environments. Nevertheless, the proposed MetaMamba provides a useful technical reference for ecological management and conservation in desert rangeland.

## Conclusions

To address the problem of limited labeled samples in desert rangeland, this study proposes a MetaMamba classification method based on a meta-learning framework. The proposed method constructs a dual-branch structure consisting of local and global branches in the feature extraction stage. The local branch uses CNNs to extract fine-grained spatial features, while the global branch models long-range spatial dependencies based on the Mamba model. On this basis, combined with a meta-learning strategy, the model can learn feature representations with strong generalization ability under few-shot conditions. Experimental results show that MetaMamba achieves better overall classification performance than comparison methods. Meanwhile, it demonstrates good stability and adaptability under different training sample sizes. Ablation studies further verify the important role of local-global feature fusion in improving model performance. The feature visualization results show that the representations learned by MetaMamba are more discriminative and can effectively reduce confusion between different vegetation classes. Overall, the proposed method provides an effective technical approach for vegetation monitoring and ecological assessment in desert rangeland.

### Code Availability

The source code used in this study is publicly available on GitHub: https://github.com/zhang2508/MetaMamba.
